# Virtual Reality-Induced Modification of Vestibulo–Ocular Reflex Gain in Posturography Tests

**DOI:** 10.3390/jcm13102742

**Published:** 2024-05-07

**Authors:** Jan Warchoł, Anna Tetych, Robert Tomaszewski, Bartłomiej Kowalczyk, Grażyna Olchowik

**Affiliations:** 1 Department of Biophysics, Medical University of Lublin, K. Jaczewskiego 4, 20-090 Lublin, Poland; anna.tetych@umlub.pl (A.T.); bartlomiej.kowalczyk@umlub.pl (B.K.); olchowikgrazyna@gmail.com (G.O.); 2 Department of Computer Science, University of Applied Sciences in Biala Podlaska, Sidorska 95/97, 21-500 Biala Podlaska, Poland; r.tomaszewski@dyd.akademiabialska.pl

**Keywords:** virtual reality, posturography, vestibulo–ocular reflex, optokinetic reflex, Sensory Organization Test

## Abstract

**Background**: The aim of the study was to demonstrate the influence of virtual reality (VR) exposure on postural stability and determine the mechanism of this influence. **Methods**: Twenty-six male participants aged 21–23 years were included, who underwent postural stability assessment twice before and after a few minute of single VR exposure. The VR projection was a computer-generated simulation of the surrounding scenery. Postural stability was assessed using the Sensory Organization Test (SOT), using Computerized Dynamic Posturography (CDP). **Results**: The findings indicated that VR exposure affects the visual and vestibular systems. Significant differences (*p* < 0.05) in results before and after VR exposure were observed in tests on an unstable surface. It was confirmed that VR exposure has a positive influence on postural stability, attributed to an increase in the sensory weight of the vestibular system. Partial evidence suggested that the reduction in vestibulo-ocular reflex (VOR) reinforcement may result in an adaptive shift to the optokinetic reflex (OKR). **Conclusions**: By modifying the process of environmental perception through artificial sensory simulation, the influence of VR on postural stability has been demonstrated. The validity of this type of research is determined by the effectiveness of VR techniques in the field of vestibular rehabilitation.

## 1. Introduction

Body balance and postural reactions are essential components of a healthy lifestyle and precise interaction with the surrounding environment. Balance control is a complex process involving the integration of sensory inputs from the vestibular, visual, and somatosensory systems. Maintaining balance requires strict coordination of these control systems [1]. However, their usage is not uniform. Postural stability relies on a balance between the “weights” of the three types of sensory inputs and the accurate processing of signals transmitted from the central nervous system to the motor organs [2].

Adapting spatial information from different sensory sources and the ability to transform sensory signals into motor commands require proper coordination of visual-motor skills. Adaptation can occur to achieve the goal of movement after exposure to visual signals, which is conditioned by the plasticity of the processes involved. Since sensory effects are additive [3], adaptive shifting between sensory signal sources is reasonable to achieve full visual-motor coordination.

In the case of a stable support surface and a stabilized body posture, the somatosensory system plays the most significant role in maintaining balance. The situation changes on an unstable surface or during head movement. Then, the processing of spatial orientation information needed for body balancing shifts in favor of visual cues [4].

Using the visual system to maintain balance requires good visual acuity. In addition to the optical system of the eye, efficient operation of the visual stabilization system is necessary. The vestibulo–ocular reflex (VOR) is a stabilizing reflex that acts on images formed on the retina due to the activation of the semicircular canals of the vestibular system during head movement [5]. The gaze is kept stable, causing eye movements in the opposite direction to the head movement [6]. Stabilizing eye movements can occur independently of image formation on the retina. However, if an image is formed, an additional effect is the fixation of the gaze on the observed object [7].

Head movement also causes a shift of the entire image on the retina within the field of view, stimulating optokinetic eye movements. This is the optokinetic reflex (OKR), complementing the vestibulo–ocular reflex during prolonged, slow head movements [8]. Control of gaze stabilization mechanisms becomes crucial, with clear visual information being necessary for sharp object imaging. When head movement and object movement in the field of view cause the image to be formed outside the central foveal pit, it becomes blurry. This serves as a trigger for OKR activation. Another issue is the formation of a single stereoscopic image, influenced by vergence movements combined with accommodation, depending on whether the observed object changes its distance from the observer [9].

Since there is delicate involuntary head movement even when a person is still, a reflexive eye movement is required to maintain stable vision and maximize visual potential. However, if a person makes rapid movements or an attention-attracting object appears in the field of view, saccadic movements occur additionally [10]. These are associated with rapid shifts of the visual line to points in the visual environment that provide information necessary for the current vision. However, this process involves a relatively large delay. The vision process is not continuous but rather periodic, extracting information from the environment periodically [4]. The information obtained in this way is integrated into the central nervous system, creating a visual impression.

The interaction between vestibulo–ocular and optokinetic reflexes, as well as other eye movements, occurs through the activity of the reticular formation, cerebellum, and other central nervous system centers. This allows for the inhibition of vestibular nucleus activity by cortical centers responsible for eye movements [11]. Thus, the mismatch between visual and vestibular information can modify the reinforcement of the vestibulo–ocular reflex (VOR).

Based on the theoretical analysis of the topic and the planned research, the authors of the study proposed two hypotheses for consideration. The first one is whether exposure of the patient to VR affects postural stability by changing the sensory weighting of the vestibular system. The other one is whether the reduction in VOR reinforcement results in adaptive shifting to OKR.

## 2. Materials and Methods

### 2.1. Recruitment and Selection

The study was conducted on a group of 40 men aged 21–23 years (mean 21.38 ± 0.64 years) with heights ranging from 173 to 196 cm (mean 183.46 ± 6.50 cm). Participants were physiotherapy students at the Medical University of Lublin. Each participant was briefed on the sequence of examinations and completed a health questionnaire prior to participation, providing informed written consent. The study procedures were approved by the Ethics Committee of the Medical University of Lublin on 28 November 2019, approval number 0254/317/2019.

Based on a proprietary health assessment questionnaire, 14 individuals were excluded from analysis if they reported the following: postural defects—3 individuals; uncorrected visual impairments—5 individuals; ongoing medication use—2 individuals; dizziness and balance disorders—1 individual; head and cervical spine injuries—1 individual; chronic illnesses—1 individual; and individuals who reported consuming alcohol and caffeine-containing beverages within 24 h prior to the study—1 individual.

Finally, 26 young men participated in the study. The research protocol for the selected group included the following steps: Sensory Organization Test (SOT), VR exposure in a seated position, followed by another SOT test after exposure. At no point did participants report any discomfort.

### 2.2. Measurement Procedure

To assess the postural stability of the participants, the Computerized Dynamic Posturography (CDP) method was chosen, utilizing the NeuroCom—Balance Manager^®^ posturography device. This method evaluates changes in the body’s center of gravity (COG) induced by the introduction of stimuli that may disrupt the functioning of one of the three existing and cooperating balance systems in our body: the somatosensory, visual, and vestibular systems. Changes in COG position are recorded by load cells placed in the platform on which the participant stands.

To conduct the analysis of postural stability, SOT and Sensory Analysis were chosen from among the available tests and research protocols.

The SOT quantitatively assesses the ability of the participant to select appropriate sensory inputs of the sensory system by distinguishing the influence of individual signals on postural response [12]. The values obtained by the participants in this test ranged from 0 to 100 percent. A score of 100% indicates that the participant’s COG did not move anteriorly or posteriorly relative to the theoretical maximum displacement. The lower the percentage value of the result, the greater the displacement, and a value of 0 indicates that the participant’s sway amplitude approaches the limits of stability. The SOT test is conducted under 6 conditions:

SOT1—eyes open, stable platform, static surroundings, evaluating the somatosensory system;
SOT2—eyes closed, stable platform, static surroundings, evaluating the somatosensory system;
SOT3—eyes open, stable platform, dynamic surroundings, evaluating the somatosensory system;
SOT4—eyes open, moving platform, static surroundings, evaluating the visual system;
SOT5—eyes closed, moving platform, static surroundings, evaluating the vestibular system;
SOT6—eyes open, moving platform, dynamic surroundings, evaluating the vestibular system.

Each SOT test was repeated three times during the study, and the result obtained is the arithmetic mean of the SOT tests. The result of this mean is described as the Equilibrium Score (ES) with designations identical to those in the SOT test, ranging from ES1 to ES6. Additionally, a weighted average is calculated from tests ES1 to ES6, named the Composite Equilibrium Score (CES).

During the SOT test, Sensory Analysis was also conducted. It directly assesses the effect of introducing disruptive stimuli for one of the three balance systems:

SOM = ES2/ES1—somatosensory system;
VIS = ES4/ES1—visual system;
VEST = ES5/ES1—vestibular system;
PREF = (ES3 + ES6)/(ES2 + ES5)—visual preferences, indicating the patient’s inclination to choose visual signals, even if they are disrupted.

**Figure 1:**
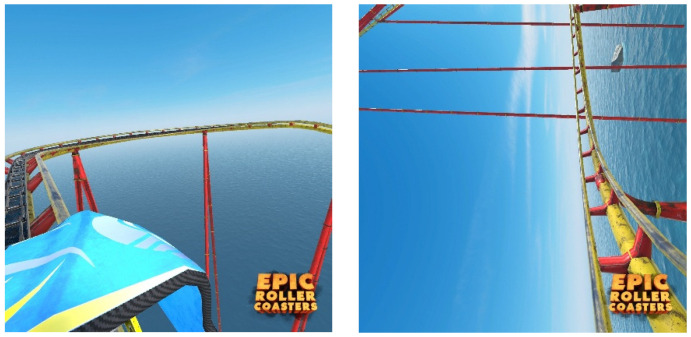
Screenshots from the Mini World Roller Coaster application.

To expose the male participants to VR, equipment from HTC, the Vive model, was used. The set comprises an HMD (Head Mounted Display) with two built-in OLED screens with a resolution of 1080 × 1200 pixels and a refresh rate of 90 Hz each, providing a combined field of view of 110°. Additional components of the set include 2 motion controllers and 2 laser emitter devices called Lighthouse, capable of tracking movement in 6 axes. The image source delivered to the HMD goggles was a Dell Precision Tower computer operating on a 64-bit Windows 10 Pro operating system, equipped with an Intel Core TM I7-7700 3.60 GHz CPU and 32 GB RAM. The image used for exposure was the VR Mini World Roller Coaster (Figure 1) with sound transmission disabled.

### 2.3. Statistical Analysis

The statistical analysis of the obtained survey results and data from the posturography study was conducted using the Statistica 13.3 software. The Shapiro–Wilk test showed a lack of normal distribution, so all statistics were based on the median, along with the upper and lower quartiles. The significance level in the analyses was set at *p* < 0.05. To determine whether significant changes occurred in posturography results after subjecting participants to VR exposure compared to before, the Wilcoxon signed-rank test was employed.

## 3. Results

**Figure 2:**
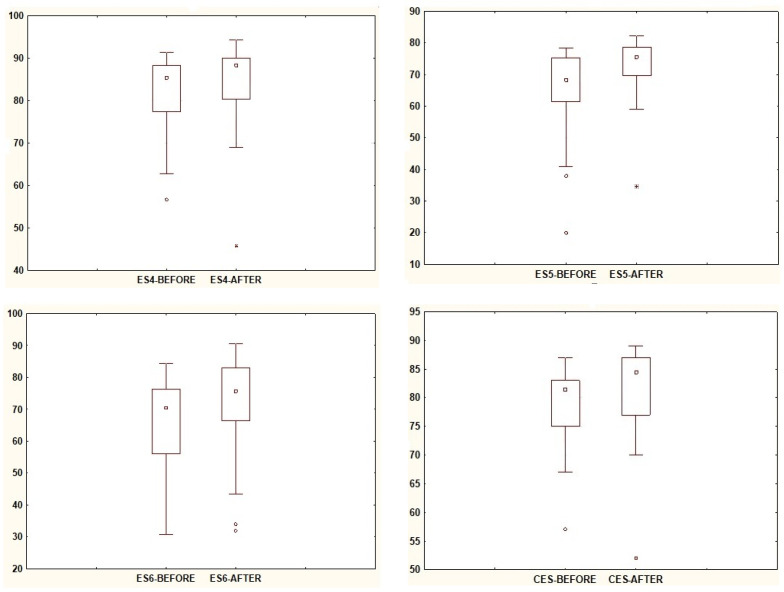
The results of statistically significant parameters ES4, ES5, ES6, and CES. Note: square—median; box—interquartile range (IRQ = Q3–Q1); whiskers—maximum and minimum values; dot—outliers; star—extreme.

**Figure 3:**
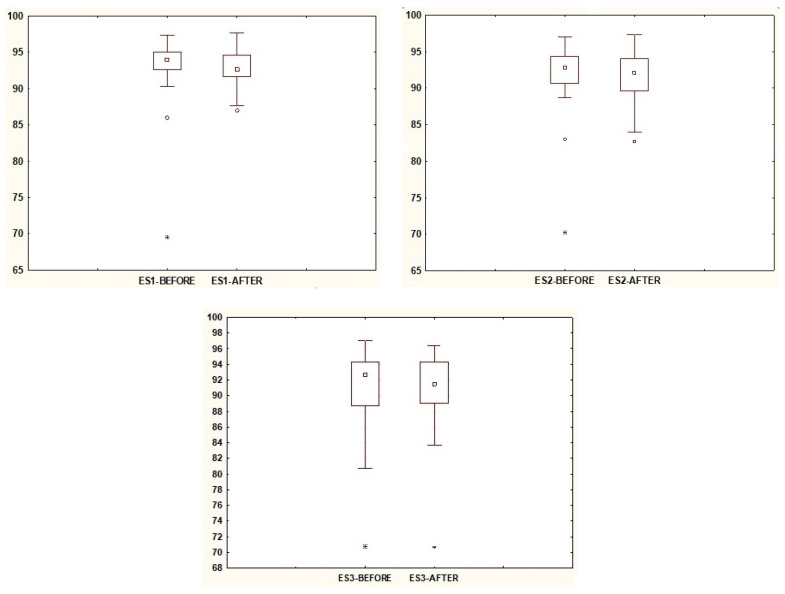
The results of statistically insignificant parameters ES1, ES2, and ES3. Note: square—median; box—interquartile range (IRQ = Q3–Q1); whiskers—maximum and minimum values; dot—outliers; star—extreme.

In Table 1, the relationships between the posturography test results in the SOT before VR exposure and the results obtained in the SOT after VR exposure are presented. The comparison of median scores clearly indicates the influence of VR on the sensory response of the balance system. Statistical significance was demonstrated in trials ES4, ES5, and ES6 (Figure 2), where dynamic disturbances of sensory input signals occurred in each case. The interquartile range in trial ES5 is smaller, indicating a narrower spread of results. The trial results also showed statistical significance for the overall balance score, CES, which is lower before VR exposure and higher afterward. Regarding trials studied on the posturography platform in a fixed position (i.e., trials ES1, ES2, and ES3), the median values decreased after VR exposure, but these changes were not statistically significant (Figure 3).

**Figure 4:**
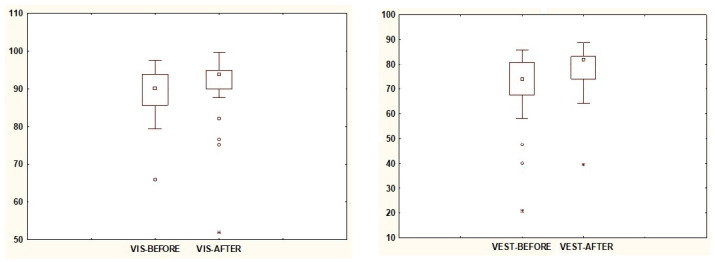
The results of statistically significant parameters VIS and VEST. Note: square—median; box—interquartile range (IRQ = Q3–Q1); whiskers—maximum and minimum values; dot—outliers; star—extreme.

**Figure 5:**
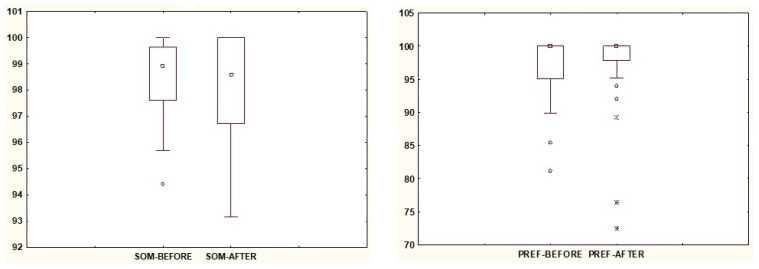
The results of statistically insignificant parameters SOM and PREF. Note: square—median; box—interquartile range (IRQ = Q3–Q1); whiskers—maximum and minimum values; dot—outliers, a star—extreme.

In Table 2, the relationships between the results of Sensory Analysis of posturography before VR exposure and the results obtained after exposure are presented. Based on the median, it was shown that the value of the SOM parameter, indicating the usefulness of signals from the somatosensory system, is lower after VR exposure. Statistical significance was demonstrated for the VEST and VIS parameters (Figure 4), whose numerical values increased after exposure. The value of PREF remained unchanged after subjecting the participants to VR exposure, confirming the same median value in both cases, while the spread of values decreased after exposure (Figure 5).

## 4. Discussion

The obtained research results unequivocally indicate the influence of VR on the vestibular and visual systems. This influence is the result of modifying the perception of the environment through artificial sensory stimulation. At the physiological core of balance maintenance lies a delicate balance of processes in processing receptor stimuli into postural responses. Perception of reality is based on static and dynamic spatial information perceived by our senses. However, in the case of VR presentation, the images on the HMD display and the corresponding information about head position and movements differ [13], causing a visual–vestibular conflict (VVC) [14]. In Akizuki’s studies [14,15], there is an indication of the possibility of inducing motion sickness and postural instability due to conflicting visual and vestibular afferent stimuli. Objective signs of instability were observed after stimulating the visual–vestibular conflict (VVC) using VR. Nishike et al. [16] expanded the scope of research on the influence of sensory stimuli on the visual–vestibular somatic conflict induced by the VR environment. They suggested that adaptation in a conflicted state reduces the contribution of visual stimuli in posture control by altering the weight of vestibular–somatosensory stimuli. Conversely, Girolamo [4] points to an adaptation disrupting the eye-head coordination strategy by modifying the vestibulospinal reflex (VSR), manifested by impaired postural control during VR exposure.

The research conducted by the authors of this study leads to similar conclusions. In the results of ES1, ES2, and ES3, the median values are lower after VR exposure. This is the first suggestion that VR influences the vestibular system. These tests are conducted on a stable surface, where up to 70% of processed receptor stimuli originate from the proprioceptive system. Because there are no direct mechanisms connecting the visual and somatosensory systems, this indicates such a strong influence of VR on the vestibular system that, despite the paradigm suggesting its maximum use of 20% [12], disturbance in the stability of the subjects is evident in the first three tests. The result is similar to that of Nishike’s research [16]. This indicates a change in the sensory weight of the vestibular system, causing a conflict of sensory information. However, it is intriguing that this effect was observed after the VR exposure ended. This type of instability was described by Cobb [17]. These were studies on the effects of altered or simulated visual environments. It was shown that individuals subjected to conditions of disturbed visual environment may experience postural instability effects upon return to a natural environment [18]. According to the authors [17], the effect is mild and short-lived, as confirmed by the results of the authors’ own research in this study. No statistical significance was found in the differences between tests on a stable surface before and after VR exposure, which may indicate a mild influence of VR on the postural stability of the subjects in the SOT1, SOT2, and SOT3 tests.

In Horlings et al.’s research [19], it was shown that in individuals standing on a stable surface, VR exposure leads to an increase in postural sway amplitude similar to that caused by eye closure. In the authors’ own studies, this corresponds to the conditions of SOT2. The same effect of increased sway amplitude observed by Horlings was also observed on a foam surface, indicating instability [19]. His research on the influence of VR on postural stability was conducted in real time. In the authors’ own studies, it is therefore puzzling why patients achieve better results in postural response tests on an unstable surface (SOT4, SOT5, and SOT6 tests) after VR exposure. In this situation, even 60% of sensory information comes from the vestibular system, with approximately 30% being visual stimuli [12]. The differences in results between ES4, ES5, and ES6 before and after VR exposure are also statistically significant. The situation is similar for CES, which is an indicator of overall body balance, considering the displacement of the organism’s COG in all SOT conditions. The authors of this study attempted to explain this result.

Since virtual reality (VR) only simulates visual processes, it is reasonable to provide a deeper explanation of the correlation between visual and vestibular perception. One of the connections is the vestibulo–ocular reflex (VOR). Its role is to stabilize the image on the retina during rapid head movement [20]. However, during VR presentation, the patient does not utilize vestibular information in the same way as when looking at real objects. The delay resulting from the analysis of the patient’s head position with the HMD, according to the manufacturers’ assurances, is minimal, around 20 ms. However, in reality, it can reach up to 50 ms [21], which is much longer than the VOR latency. This creates a difference in “sensory weighting” due to the mismatch between visual and vestibular information (VVC). The nervous system adapts to eliminate the distortion of the sense of movement [14].

Weech and Troje [22] found that to resolve this sensory conflict, the effectiveness of vestibular stimuli decreases. This manifests as a change in the reinforcement of the vestibulo–ocular reflex (VOR). It becomes an unnecessary burden on the nervous system, which may cause an adaptive shift in favor of other mechanisms stabilizing the image on the retina [4]. The adaptation process itself is possible due to changes in the activity of vestibular nuclei [11]. According to Bonnet [23], the central nervous system (CNS) must create synergies between visual and postural signals to carry out precise visual tasks. In Bonnet’s research [23], it was demonstrated that the CNS does not treat body movements as the main goal of the visual pursuit of exposed fixation areas but adjusts postural reactions to oculomotor activities. However, synergy does not occur without an increase in sensory load. When the CNS load is maintained at a constant level, there is no correlation between oculomotor and postural behaviors in precise visual tasks, and visual–postural synergy is not associated with changes in sensory load. In such cases, distracting environmental stimuli, such as visual signals, may be processed less rigorously [24]. Therefore, the level of perceptual load is an important factor when focusing the attention of the subjects on specific stimuli.

The choice of VR presentation in our studies in the form of a roller coaster was motivated by the fact that each participant received the same type and amount of distracting stimuli. The position of objects was determined by the path traveled in virtual reality. Thus, conditions conducive to visual fixation prevailed during VR projection. To minimize the number of stimuli other than visual ones, auditory feedback during the presentation was turned off.

The angular range of the device used in the studies is approximately 110 degrees. Research conducted by Emmerik [25] indicates that for medical VR applications, a field of view of this magnitude is sufficient. Above this value, the image may appear unnatural to the observer and may induce cyber sickness [26]. The displayed image is dynamic, thus involving changes in the position of the optical signal. Human perception is precise only within a 1.5-degree field of view, so there are several areas on the retina associated with the perception and evaluation of new objects, spatial orientation, and, consequently, the balance system. The central, parafoveal, and peripheral parts of the visual field determine differences in retinal structure and, therefore, the involvement of heterogeneous brain structures in processing specific information from these areas [27]. The farther from the foveal area, the less sharp the image [28]. However, for the space in front of us to be clearly perceived, the visual system must continuously analyze the entire field of view and, at a frequency of several Hz, decide on object selection, directing gaze toward it, coordinating temporally, and determining the spatial parameters of movement. Therefore, reflexes enabling visual sensation stabilization are necessary.

In addition to VOR is OKR [10], which compensates for head movements to maintain fixation on a selected object, and shifting adaptation to it seems most logical. The refresh rate of the displayed image in the device used in our studies is 90 Hz, indicating changes faster than the OKR latency time (50–100 ms). However, the activation conditions of OKR are sufficient because the speed of object position changes on the display is limited by the time it takes for the computer to process the signal from the VR device’s position sensors on the patient’s head, as well as the inertia of the sensors themselves [21]. 

During visual fixation, the eyes also do not remain stationary; eye movement is carried out even without engaging visual attention [29].

Upon completion of VR exposure, control of postural response is once again supplemented by both visual and vestibular signals. Increasing the reinforcement of VOR may then cause excessive eye movements, leading to the slipping of the entire image on the retina. This, in turn, serves as the best stimulus, triggering the optokinetic reflex OKR to increase image stability [10]. The precision of visual stimulus reception consequently results in increased postural stability. This is confirmed by the higher values of ES4 and VIS, which may be the result of an adaptive shift from VOR to OKR. However, this does not fully explain the positive influence of VR on postural stability in the examination of an unstable surface. Visual stimuli in posturographic studies do not match the dynamics of virtual reality images. Considering the test conditions of SOT5 may help explain this phenomenon. It is performed on an unstable surface with closed eyes [12], which does not hinder the triggering of the VOR [30]. If prolonged adaptive shifts are recorded after VR exposure, it may also indicate a prolonged reduction in VOR reinforcement. This occurs due to the re-adaptation of the vestibular system [4], benefiting its sensory weight and thus increasing body stability in SOT5 conditions. This is confirmed by the values of ES5 and VEST, which are higher after VR exposure than before exposure.

Another confirmation of the adaptive shift is the higher ES6 scores after VR exposure than before exposure. The patient’s moving environment does not require rapid head movements. Fixation and the optokinetic reflex OKR may then play a crucial role in stabilizing the image on the retina, assisting the process of visual fixation [31].

It is also interesting to note the improvement in patient stability despite the disturbance of visual signals causing emergency movements [32]. In the ES6 test, there is a change in the distance of the visual environment. While VR projection, image fusion, and single vision result from the perceptual adaptation of the nervous system [33]. In the SOT6 test, proper stereoscopic vision significantly influences postural stability. Changing the distance of the visual environment from the eye also involves changes in accommodation, but several studies have shown that only convergence movements provide a cue for distance assessment [34]. It has been shown that the influence of visual preferences (PREF) did not change after VR exposure, indicating that it did not cause significant changes in the patient’s dependence on visual stimuli. In both cases, the visual system received disturbed visual information, resulting in increased sensory load. According to McGeehan’s research [35], this leads to an increased importance of vestibular balance control. This expanded neuronal strategy may act to supplement the limited cortical processing resources in the balance system. This is also another fact indicating the possibility of adaptive shifts benefiting the sensory weight of the vestibular organ. The process of exposing patients to VR can be regarded as an additional sensory test for this organ [36].

The positive impact of VR on postural response is likely to be temporary in nature. According to authors [4], it takes about 30 min. However, the process of nervous system adaptation through VOR calibration triggers neuronal plasticity, resulting in long-lasting synaptic changes in vestibular nuclei [37]. This is due to the neuronal mechanisms underlying motor learning required for VOR adaptation and compensation [20]. This suggests the potential use of VR techniques in the therapy of individuals with disorders involving vestibular dysfunction. Based on clinical studies and case reports, it can be assumed that the optimal use of VR for patient rehabilitation may be combined with conventional therapies [38]. The advantage of VR over other neurotherapy techniques lies in the greater variety of stimuli [39]. It is also possible to use the HMD device itself as an alternative to posturographs [40], although full replication of balance behavior on an unstable surface is not possible in this case.

Various VR systems and environments facilitate personalization and adaptation of treatment for vestibular disorders, as they allow for the simulation of different tasks with specific therapeutic goals [41]. Research indicates that VR devices can be used as effective tools to motivate patients during rehabilitation sessions, improve spatial orientation, and maintain balance, thereby enhancing the functionality of patients in daily activities [42]. However, tasks presented in the VR environment should be prepared at various levels of difficulty and intensity, taking into account differences in the tolerance levels of individual persons [24].

## 5. Conclusions

By modifying the process of environmental perception through artificial sensory simulation, the influence of VR on postural stability has been demonstrated. The research results suggest that this is due to a change in the sensory weighting of the vestibular system. Therefore, this confirms the first hypothesis posed in this study.

Based on the analysis of visual perception conditions and postural responses in the subjects, the modification of VOR reinforcement was confirmed, and the possibility of adaptive shifting to OKR was demonstrated. Thus, the other hypothesis was partially confirmed.

Extended effects of VR exposure on the subjects were also demonstrated, which may confirm neuronal plasticity due to synaptic changes in vestibular nuclei. Due to the increasing popularity of VR imaging techniques, further research on their influence on stability is necessary. It is justified to continue research also within the group of women to assess the effect of gender on the effectiveness of therapy using VR. However, differentiating the results between male and female groups necessitates increasing the group size.

## Figures and Tables

**Table 1:** Sensory Organization Test (SOT)—Equilibrium Score (ES) and Composite Equilibrium Score (CES) (ES scores correspond to individual SOT conditions). Note: * *p* < 0.05—statistically significant result.

	Before VR (26)	After VR (26)	
Test	Med (IRQ)	Med (IRQ)	*p*
ES1 [%]	94.00 (2.33)	92.67 (3.00)	0.21
ES2 [%]	92.83 (3.67)	92.17 (4.33)	0.13
ES3 [%]	92.67 (5.67)	91.50 (5.33)	0.35
ES4 [%]	85.33 (11.00)	88.33 (9.67)	0.03 *
ES5 [%]	68.33 (14.00)	75.50 (9.00)	0.01 *
ES6 [%]	70.50 (20.33)	75.67 (16.67)	0.01 *
CES [%]	81.50 (8.00)	84.5 (10.00)	0.03 *

**Table 2:** Results of Sensory Analysis research. Note: * *p* < 0.05—statistically significant result.

	Before VR (26)	After VR (26)	
Test	Med (IRQ)	Med (IRQ)	*p*
SOM [%]	98.93 (2.05)	98.60 (3.27)	0.29
VIS [%]	90.07 (8.23)	93.82 (5.04)	0.05 *
VEST [%]	74.15 (13.11)	81.81 (9.03)	0.02 *
PREF [%]	100.00 (4.94)	100.00 (2.18)	0.86

## Data Availability

The data supporting reported results are available in the corresponding author.
